# Supplementary material for: A Plasmid-Based Fluorescence Reporter System for Monitoring Oxidative Damage in E. coli
Source: Sensors (Basel). 2022 Aug 23;22(17):6334. doi: 10.3390/s22176334 (PMC9459809; doi:10.3390/s22176334)
Supplement: Supplementary file 1 [file sensors-22-06334-s001.zip › sensors-1850049-supplementary.pdf]

Supplementary Material

A plasmid-based fluorescence reporter system for monitoring oxidative damage in *E. coli*

Hariharan Dandapani, Pasi Kankaanpää, Patrik R. Jones, Pauli Kallio*.

* Corresponding author. Email: pataka@utu.fi

This file includes:

Supplementary Figure S1: DNA sequence of the IscR-C reporter cassette used in this study. Page 2.

Supplementary Figure S2: Growth curves of DH5α and BW25113 harbouring pJ404-IscR-C. Page 3

Supplementary Figure S3: Fluorescence response without OD_{600nm} normalisation of the sensor systems towards oxidative stress induced by 2 mmol l^{-1} supplemented H_2O_2. Page 4.

Supplementary Figure S4. LB plates of *E. coli* DH5α with pJ404-empty and pJ404-IscR-C. Page 5.

Supplementary Figure S5. Fluorescence response without OD_{600nm} normalisation of the sensor system towards lowered cultivation temperature. Page 6.

Supplementary Figure S6. Representative confocal microscopy images of *E. coli* reporter strain DH5α/ pJ404-IscR-C grown at 37°C with and without 2 mmol l^{-1} supplemented H_2O_2. Page 7.

GAGCTCAGGAGGTAAAAAAATGCGCCTGACGTCCAAGGGCCGCTACGCTGTAACCGCCATGCTGGACG
TGGCCCTGAATAGCGAAGCAGGTCCGGTTCCGCTGGCAGACATTAGCGAGCGTCAAGGCATTTCCCTG
AGCTATCTGGAGCAATTGTTTAGCCGTCTGCGTAAGAATGGCCTGGTCAGCTCGGTGCGTGGTCCGGG
TGGCGGCTACCTGCTGGGCAAAGATGCGAGCAGCATCGCTGTCGGTGAAGTCATCAGCGCGGTCGATG
AAAGCGTTGATGCAACCCGTTGCCAGGGTAAAGGTGGTTGTCAGGGTGGTGATAAATGCCTGACGCAC
GCGTTGTGGCGCGACCTGAGCGACCGTCTGACCGGTTTCTTGAACAACATCACCCTGGGTGAGTTGGT
GAACAATCAAGAGGTGCTGGATGTTTCTGGCCGTCAGCATACCCACGATGCGCCACGTACTCGCACGC
AGGACGCGATTGACGTTAAGCTGCGCGCGTAAGAGCTCGGATCCATGCATAGGTTAAATACCCGACTA
AATCAGTCAAGTAAATAGTTGACCAATTTACTCGGGAATGTCAGACTTGACCCTGCTATGCATATACC
CCCACTTTTACAATAAAAAACCCCGGGCAGGGGCGAGTTTGAGGTGAAGTAAGACCATGGATAGCACT
GAGAACGTCATCAAGCCCTTCATGCGCTTCAAGGTGCACATGGAGGGCTCCGTGAACGGCCACGAGTT
CGAGATCGAGGGCGTGGGCGAGGGCAAGCCCTACGAGGGCACCCAGACCGCCAAGCTGCAAGTGACCA
AGGGCGGCCCCCTGCCCTTCGCCTGGGACATCCTGTCCCCCCAGTTCTTCTACGGCTCCAAGGCGTAC
ATCAAGCACCCCGCCGACATCCCCGACTACCTCAAGCAGTCCTTCCCCGAGGGCTTCAAGTGGGAGCG
CGTGATGAACTTCGAGGACGGCGGCGTGGTGACCGTGACCCAGGACTCCTCCCTGCAGGACGGCACCC
TCATCTACCACGTGAAGTTCATCGGCGTGAACTTCCCCTCCGACGGCCCCGTAATGCAGAAGAAGACT
CTGGGCTGGGAGCCCTCCACTGAGCGCAACTACCCCCGCGACGGCGTGCTGAAGGGCGAGAACCACAT
GGCGCTGAAGCTGAAGGGCGGCGGCCACTACCTGTGTGAGTTCAAGTCCATCTACATGGCCAAGAAGC
CCGTGAAGCTGCCCGGCTACCACTACGTGGACTACAAGCTCGACATCACCTCCCACAACGAGGACTAC
ACCGTGGTGGAGCAGTACGAGCGCGCCGAGGCCCGCCACCACCTGTTCCAGAGCCACGGCTTCCCGCC
GGCGGTGGCGGCGCAGGATGATGGCACGCTGCCCATGTCTTGTGCCCAGGAGAGCGGGATGGACCGTC
ACCCTGCAGCCTGTGCTTCTGCTAGGATCAATGTGTAGGAATTC

Supplementary Figure S1: Nucleotide sequence (5'-3') of the synthesised IscR-C reporter cassette composed of the *iscR* gene (green sequence) encoding the ROS-sensitive transcriptional regulator IscR, the P_{iscR} promoter (blue sequence), the downstream reporter gene encoding the E2-Crimson fluorescent protein (red sequence), and the PEST sequence to destabilise the protein (purple sequence). Restriction sites have been underlined. The fragment was subcloned in the pJ404 backbone (EcoRI-SacI) to generate pJ404-IscR-C shown in Figure 1.

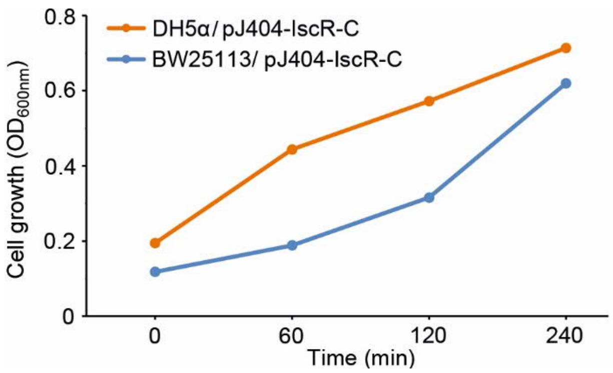

Supplementary Figure S2: **Growth curves of DH5α and BW25113 cultures harbouring pJ404-IscR-C**. The optical density (OD_{600nm}) values were measured at one-hour intervals for the strains DH5α (orange line) and BW25113 (blue line) harboring the plasmid pJ404-IscR-C. The averages and standard deviations were calculated based on three independent parallel cultures with three technical replicates each (*n*=9). Due to small standard deviations, the error bars are not always visible.

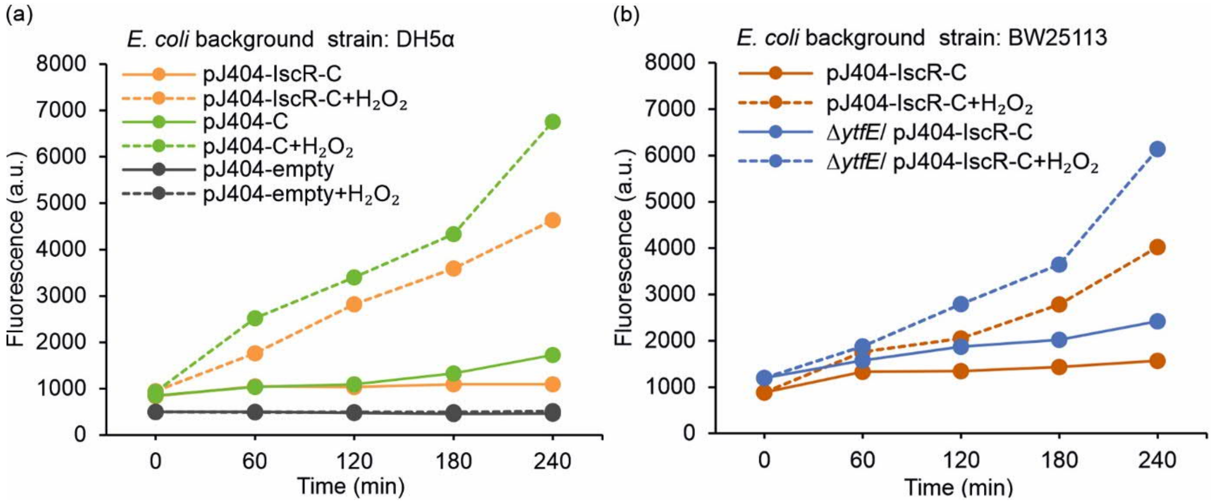

Supplementary Figure S3. Fluorescence response without OD_{600nm} normalisation of the alternative redox sensor systems towards oxidative stress induced by 2 mmol l^{-1} supplemented H_2O_2 in (**a**) DH5α and (**b**) BW25113 background (see Table 1 for strain details). The fluorescence values were measured at one-hour intervals from cultures repeatedly supplemented with 2 mmol l^{-1} H_2O_2 (dashed lines) and cultures without H_2O_2 addition (solid lines). The averages and standard deviations were calculated based on three independent parallel cultures with three technical replicates each (n=9). Due to small standard deviations, the error bars are not always visible.

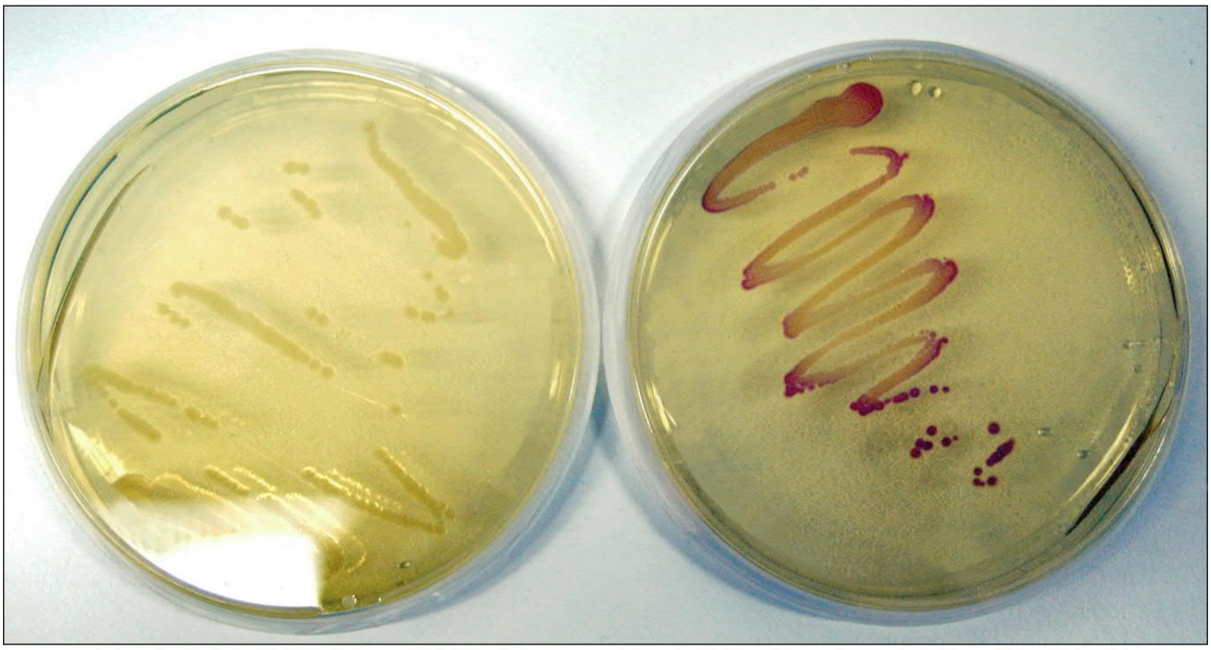

DH5α/ pJ404-empty   DH5α/ pJ404-IscR-C

Supplementary Figure S4: LB agar plates of *E. coli* DH5α harbouring the control plasmid pJ404-empty (left) and the reporter plasmid pJ404-IscR-C (right) after three-day storage in the fridge at 4ºC.

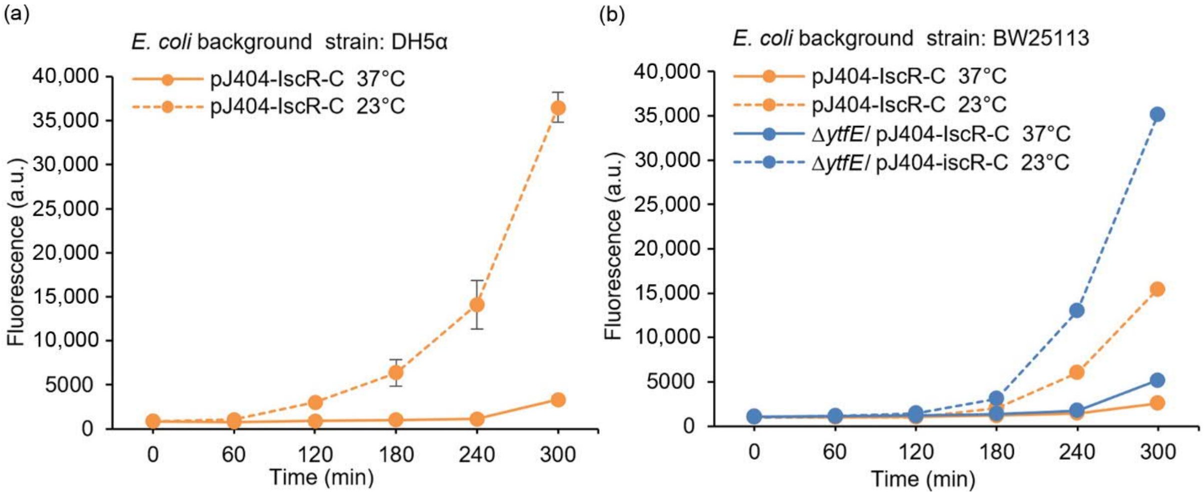

Supplementary Figure S5. Fluorescence response without OD_{600nm} normalisation of the pJ404-IscR-C sensor system towards lowered cultivation temperature in (**a**) DH5α and (**b**) BW25113 background (see Table 1 for strain details). Fluorescence was measured at one-hour intervals from cultures grown at the default temperature of 37°C (solid lines) or at 23°C (dashed line). The averages and standard deviations were calculated based on three independent parallel cultures with three technical replicates each (*n*=9). Due to small standard deviations, the error bars are not always visible.

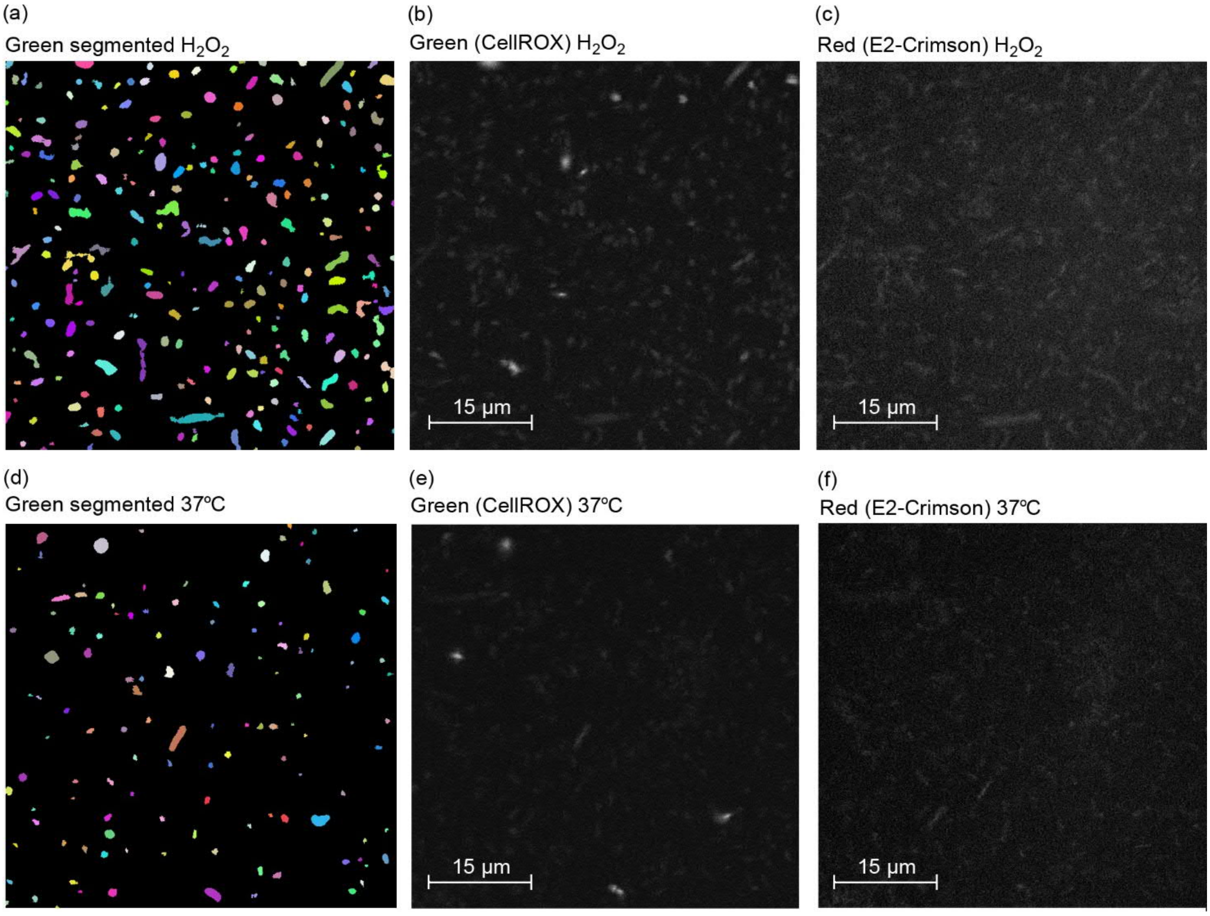

Supplementary Figure S6. Representative confocal microscopy images of *E. coli* reporter strain DH5α/ pJ404-IscR-C grown at 37°C with 2 mmol l^{-1} supplemented H_2O_2, and without H_2O_2. (**a**/**d**) green fluorescence channel after segmentation with dynamic thresholding to separate bacteria from the background, (**b**/**e**) original image of the cells emitting green fluorescence corresponding to the ROS-sensitive commercial probe CellROX, and (**c**/**f**) original image of the cells emitting red fluorescence corresponding to the IscR-regulated E2-Crimson reporter system.
